# Glioma malignancy is linked to interdependent and inverse AMOG and L1 adhesion molecule expression

**DOI:** 10.1186/s12885-019-6091-5

**Published:** 2019-09-12

**Authors:** Qiong Jiang, Qing Xie, Chengliang Hu, Zhai Yang, Peizhi Huang, Huifan Shen, Melitta Schachner, Weijiang Zhao

**Affiliations:** 1 Center for Neuroscience, Shantou University Medical College, 22 Xin Ling Road, Shantou, Guangdong 515041 People’s Republic of China; 2 Keck Center for Collaborative Neuroscience and Department of Cell Biology and Neuroscience, Rutgers University, 604 Allison Road, Piscataway, NJ 08854 USA

**Keywords:** Glioma, AMOG, L1CAM, Apoptosis, Senescence, Therapy, Human

## Abstract

**Background:**

Gliomas account for the majority of primary human brain tumors and remain a challenging neoplasm for cure due to limited therapeutic options. Cell adhesion molecules play pivotal roles in the growth and progression of glial tumors. Roles of the adhesion molecules on glia (AMOG) and L1CAM (L1) in glioma cells have been shown to correlate with tumorigenesis: Increased expression of L1 and decreased expression of AMOG correlate with degree of malignancy.

**Methods:**

We evaluated the interdependence in expression of these molecules by investigating the role of AMOG in vitro via modulation of L1 expression and analyzing apoptosis and cell senescence of glioma cells.

**Results:**

Immunohistochemical staining of normal human cortical and glioma tissue microarrays demonstrated that AMOG expression was lower in human gliomas compared to normal tissue and is inversely correlated with the degree of malignancy. Moreover, reduction of AMOG expression in human glioblastoma cells elevated L1 expression, which is accompanied by decreased cell apoptosis as well as senescence.

**Conclusion:**

AMOG and L1 interdependently regulate their expression levels not only in U-87 MG cells but also in U251 and SHG44 human glioma cell lines. The capacity of AMOG to reduce L1 expression suggests that methods for increasing AMOG expression may provide a therapeutic choice for the management of glial tumors with high expression of L1.

**Electronic supplementary material:**

The online version of this article (10.1186/s12885-019-6091-5) contains supplementary material, which is available to authorized users.

## Background

Glioma is a brain neoplasm mainly originating from glial cells. It accounts for about 30% of all tumors in the human central nervous system and about 80% of the malignant ones [1, 2]. Glial tumors are characterized by infiltrative growth behavior, high proliferative potential, intratumoral heterogeneity, and recurrence. They are graded from I to IV according to the criteria of the World Health Organization (WHO). In existing practice, treatment of gliomas mainly includes surgical resection, radiotherapy and chemotherapy. However, these regimens are often not successful in the management of gliomas and lead to recurrence and progression to malignancy. It appears, therefore, necessary to identify the molecular players involved in the development and progression of glioma malignancy [1].

Several cell adhesion molecules have been identified to underlie the occurrence of malignancies in gliomas, including adhesion molecule on glia (AMOG) [3] and neural cell adhesion molecule L1 (L1CAM, hereafter abbreviated L1) [4, 5]. AMOG was first identified as an integral membrane glycoprotein highly expressed by astrocytes and shown to mediate the interaction between neurons and astrocytes [3, 6] and thereafter identified to be the β2-subunit of Na^+^/K^+^-ATPase, based on genomic structure and cDNA sequence [7–10]. Another study had also shown that AMOG enhances neurite outgrowth of cultured cerebellar and hippocampal neurons [11]. In addition, overexpression of AMOG increases adhesion on Matrigel and decreases migration of glioma cells in vitro [12]. In human glioblastoma cell cultures captured from surgical specimens, enhanced levels of AMOG expression correlated positively with invasion without affecting migration or proliferation, and knock-down of AMOG expression promoted cell migration in cultures of human astrocytes [13].

Neural cell adhesion molecule L1 also plays a crucial role in glioma tumor progression. L1 is a transmembrane glycoprotein of the immunoglobulin superfamily and important in development, synaptic activity and regeneration after trauma [14]. Through interacting with itself and other cell adhesion molecules in homophilic and heterophilic binding mechanisms, L1 promotes glioma cell migration, invasion and metastasis. Elevated L1 expression was observed in cultured glioblastoma and neuroblastoma cells [15] who showed activation of two signaling pathways, the extrinsic and the intrinsic pathway, which are considered to be potential therapeutic targets that could lead to inhibition of development and progression of malignancy [16, 17]. Thus, insights into the molecular mechanisms underlying malignancy, apoptosis and senescence are important goals in the development of new and more effective treatments.

Based on the knowledge of L1 functions and AMOG in tumor progression, with AMOG being weakly and L1 highly expressed in high-grade gliomas, we hypothesized that there may be a functional link between AMOG and L1 expression and function in the context of glioma metastasis. We now show that down-regulation of AMOG expression is accompanied by increased L1 expression as well as cell senescence and apoptosis. Vice versa, down-regulation of L1 expression leads to increased AMOG expression. The functional link between AMOG and L1 was shown to be differentially involved in apoptosis via the AKT and ERK signaling pathways in three human glial cell lines in vitro.

## Methods

### Cell culture

The human glioblastoma U-87 MG (catalog no. CL-0238) and human glioma U251 (catalog no. CL_0237) cell lines were purchased from Procell Life Science & Technology Co., Ltd. (Wuhan, China) in April 2018. The human glioma SHG-44 (catalog no. SHG44) cell line was purchased from Guangzhou Jennio Biotech Co., Ltd. (Guangzhou, China). All cell lines were recently authenticated by short tandem repeat (STR) analysis and tested for mycoplasma contamination. After being delivered, cell lines were maintained in Dulbecco’s modified Eagle’s medium (DMEM, HyClone™; Thermo Fisher Scientific, catalog no. 11330032, Beijing, China) supplemented with 50 U/mL of a penicillin/streptomycin mixture (Solarbio, catalog no. P1400, Beijing, China) and 10% fetal bovine serum (FBS, Sijiqing Biotech, catalog no. 11011–6125, Hangzhou, China). Cells were maintained in 75-cm^2^ cell culture dishes (Jet Bio-Fil, catalog no. TCD010100, Guangzhou, China) at 37 °C in a humidified 5% carbon dioxide atmosphere.

### Reagents and tissue microarray

Recombinant human L1CAM (rL1, Sino Biological, catalog no. 10140-H08H, Beijing, China) was dissolved in PBS as the stock solution (100 μg/mL) in accordance with the manufacturer’s instructions. Control siRNA, L1 siRNA and AMOG siRNA are listed in Table 1. For over-expression of human AMOG, a commercial control plasmid (pCMV3-C-GFPSpark, Sino Biological, catalog no. CV026, Beijing, China) and a human green fluorescent protein (GFP) labeled AMOG plasmid (pCMV3-AMOG-GFPSpark, catalog no. HG15882-AG, Sino Biological) were used. Lipofectamine® 2000 reagent was obtained from Invitrogen (catalog no. 11668019) and was utilized in cell transfection following the manufacturer’s instructions. Twenty-four hours after transfection, cells were maintained for another 24 h and treated with hygromycin B (Solarbio, catalog no. 10843555001) at a concentration of 500 μg/mL for the following passages. Transfection efficiency was calculated by estimating the percentage of GFP-fluorescent cells over the numbers of all total cells (set at 100%).

**Table 1:** Sequences of control siRNA, L1 siRNA and AMOG siRNA

Reagent	Sense strand sequences	Anti-sense strand sequences
Control siRNA	5′-UUCUCCGAACGUGUCACGUTT-3′	3′-TTAAGAGGCUUGCACAGUGCA-5′
AMOG siRNA	5′-GAGCCUUACAACGACUCUATT-3′	3′-TTCUCGGAAUGUUGCUGAGAU-5′
L1 siRNA	5′-GCAUUAGUGGCCAUCCUUUTT − 3′	3′-TTCGUAAUCACCGGUAGGAAA-5′

A total of 208 cases of human cerebral tissues and one skin tissue were included in the brain glioma tissue microarray containing formalin-fixed paraffin-embedded 5-μm-thick sections from human glioma and normal cerebral cortex (US Biomax, GL2083a, Derwood, MD, USA). Brain glioma tissue microarray consists of samples from different donor tissues that had been evaluated by a certified neuropathologist and scored on a scale of I to IV based on the WHO grading system as follows: tumor-adjacent normal brain tissue and normal brain tissue (*n* = 18), pilocytic astrocytoma (WHO grade I, *n* = 23), WHO grade I-II (*n* = 19), diffuse astrocytoma (WHO grade II, *n* = 85), anaplastic astrocytoma (WHO grade III, *n* = 27), and glioblastoma (WHO grade IV, *n* = 31).

### Immunohistochemistry

The microarray was deparaffinized, rehydrated via a graded series of ethanol to PBS. Antigen retrieval was performed with 10 mmol/L citrate buffer (pH 6.0) for 40 min at 99 °C. The sections were then incubated in 3% H_2_O_2_ solution to block endogenous peroxidase activity, followed by incubation with 10% non-immune goat serum for 30 min. Sections were incubated with rabbit polyclonal anti-ATP1B2/AMOG antibody (Thermo Scientific, catalog no. PA5–26279, 1:50, Rockford, USA) overnight at 4 °C PBS and incubated with biotinylated secondary antibody, and streptavidin-peroxidase conjugate according to the manufacturer’s instructions (Zhong Shan Golden Bridge Biotechnology, catalog no. PV-9000-D, Beijing, China). Enzyme activity was developed using the AEC kit (Zhong Shan Golden Bridge Biotechnology, catalog no. ZLI-9036). The sections were mounted in aqueous mounting medium (Boster, catalog no. AR1018, Pleasanton, USA).

Integrated immunostaining intensity was evaluated by digital scanning using a flashing camera (Sony, DSC-W210,). Optical density of immunostainings was evaluated using a gel imaging system and Fluochem Software (Alpha Innotech, San Leandro, CA, USA). The integrated staining intensity was evaluated on the basis of a gray scale ranging from 0 to 255 and expressed as the fold increase over the normal brain tissue sample. Images were captured with a digital microscope (Ningbo Yongxin Optics, NOVEL, DN-10, Jiangsu, China).

### Cell senescence assay

U-87 MG cells were seeded at a density of 1 × 10^4^ cells per well in 8-well chamber slides or in 96-well cell culture plates in DMEM and 10% fetal bovine serum and allowed to settle overnight. They were then transfected with AMOG siRNA or control siRNA, and then assayed after 48 h for X-Gal staining by an overnight incubation at 37 °C, according to the manufacturer’s protocol (Beyotime Biotech, cat no. C0602). Images of 9 corresponding areas were captured light microscopically. For the measurement of β-galactosidase activity, cells were digested with red blood cell lysis buffer (cat no. C3702, Beyotime Biotech) and absorbance of X-Gal reaction product was measured with a microplate reader (Infinite M1000, Tecan, Männedorf, Switzerland).

### Effects of AMOG on L1 expression

AMOG siRNA was used to evaluate the effect of AMOG on L1 expression levels, with control siRNA used as the control. U-87 MG, U251 and SHG-44 cells were tested at 60–80% confluence. The culture medium was then aspirated, replaced with fresh medium containing 200 μL siRNA-mate complexes or 0.2 μL Lipofectamine® 2000 transfection reagent for 48 h at 10 nmol/L siRNA and thereafter maintained for 48 h.

### Effects of L1 on AMOG expression

Out three L1 siRNAs the most efficient one was tested with U-87 MG, U251 and SHG-44 cells for its effect on AMOG expression under the conditions described for AMOG siRNA. In addition, the influence of recombinant human L1 extracellular domain on AMOG expression was tested by a 48 h incubation of cells with rL1 at 0, 1.0, 2.5, 5.0 and 10 nmol/L. AMOG expression was determined by Western blot analysis and immunofluorescence staining.

### Western blot analysis

Western blot analysis was performed as described [15] with antibodies listed in Table 2. Antigens were visualized using an enhanced chemilluminescence (ECL) solution (Beyotime Biotech). The signal intensity was quantified using Image J software (version 1.48, rsb.info.nih.gov/ij/) as average densitometric value multiplied by the area (measured as the number of pixels).

**Table 2:** Antibodies used for Western blot analysis

Antibody	Species	Cat. no.	Dilutions	Manufacture
L1	mouse monoclonal	MAB777	1:1000	R&D Systems
AMOG/ATP1B2	rabbit polyclonal	PA5–26279	1:1000	Thermo Scientific
p-Akt1/2/3 (11E6)	mouse monoclonal	sc-81,433	1:1000	Santa Cruz
Akt1 (G-5)	monoclonal mouse	sc-55,523	1:1000	Santa Cruz
p-Erk1/2 (E-4)	monoclonal mouse	sc-7383	1:1000	Santa Cruz
Erk1/2 (MK1)	monoclonal mouse	sc-135,900	1:1000	Santa Cruz
Bax (P-19)	polyclonal rabbit	sc-526	1:1000	Santa Cruz
Bcl-2 (C-2)	monoclonal mouse	sc-7382	1:1000	Santa Cruz
GAPDH (G-9)	monoclonal mouse	sc-3,650,620	1:1000	Santa Cruz
β-Actin (C4)	monoclonal mouse	sc-47,778	1:1000	Santa Cruz

### Immunofluorescence staining

Cells were seeded at a density of 1 × 10^4^ cells per well in an 8-chamber plate. After an overnight incubation, the cells were treated with L1 siRNA at 0, 5, 10 or 20 nM for 48 h. Cells were then fixed with 4% formaldehyde in PBS for 15 min, blocked with normal donkey serum and incubated overnight at 4 °C with monoclonal mouse anti-human L1 antibody (R&D Systems, MAB777, 1:200) or polyclonal rabbit anti-human ATP1B2/AMOG antibody (Thermo Scientific, PA5–26279, 1:200). Corresponding secondary antibodies conjugated to Dylight™ 488 (Jackson ImmunoResearch, 715–485-150, 1: 500) and Dylight™ 594 (Jackson ImmunoResearch, 711–515-152, 1: 500). Slides were mounted in ProLong® Gold Antifade reagent with DAPI (Life Technologies, ThermoFisher Scientific, P36935). Double-immunofluorescence images were acquired with laser confocal laser microscopy (Olympus, FV-1000) in a multi-track configuration.

### Statistics

Statistical analyses were performed with SPSS17.0 software (IBM, Armonk, New York, USA) and GraphPad Prism 7 (GraphPad, Los Angeles, CA, USA). Values are expressed as means ± SEM and analyzed by one-way ANOVA with Tukey’s *post-hoc* test for dependent samples or with the Student’s *t*-test for independent samples. Differences were considered statistically significant at *p < 0.05, **p < 0.01 and ***p < 0.001.

## Results

### AMOG expression in human glioma tissue microarray

**Fig. 1:**
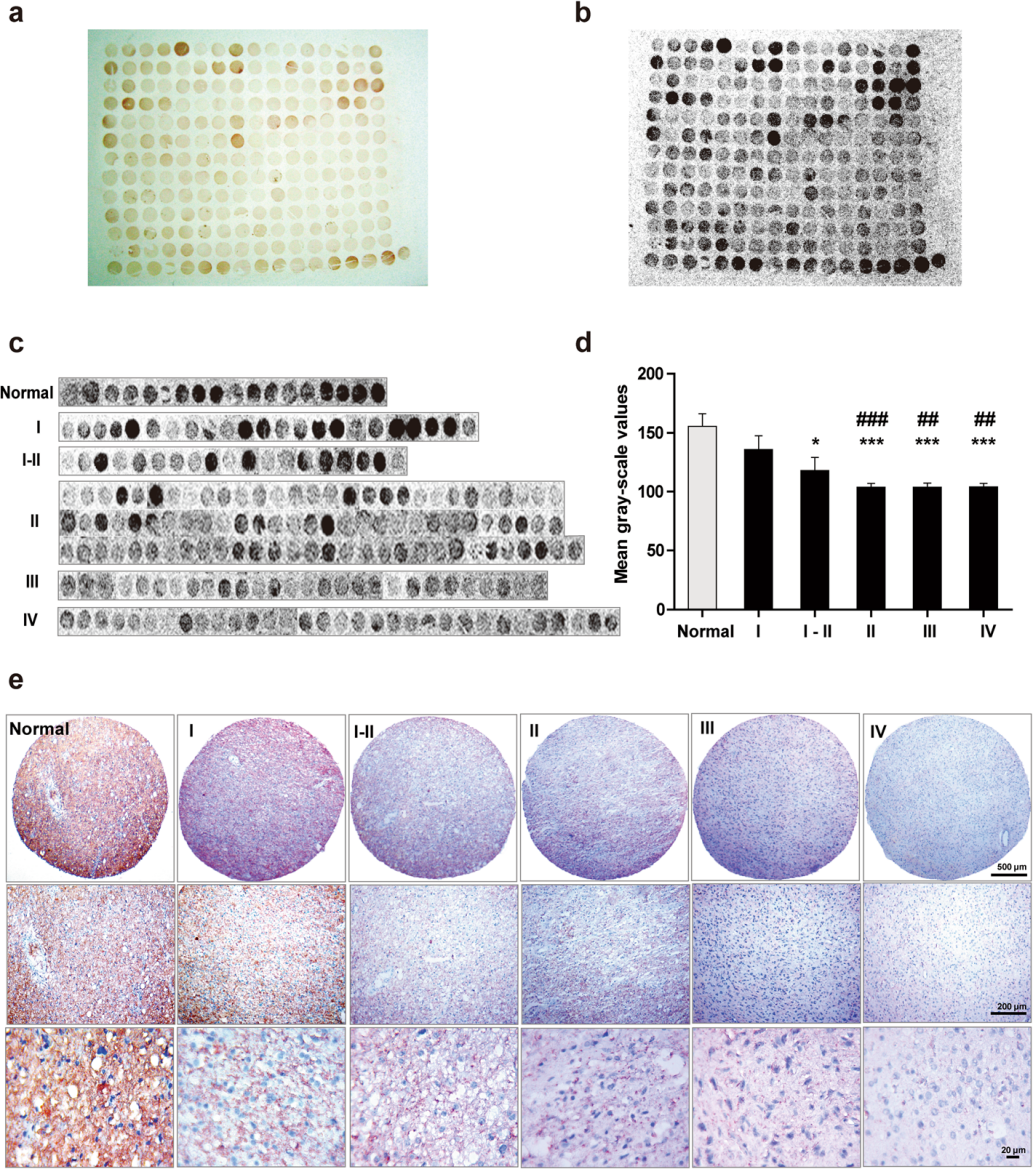
AMOG expression as assayed in a normal human brain and glioma tissue microarray. **a** Immunostaining of AMOG in the microarray containing both tumor-adjacent normal human brain and glioma tissues graded from I to IV. **b** Gray-scale image showing AMOG expression in all samples. **c** Samples from the tissue microarray were pooled in accordance with the WHO grading system. **d** Data were collected from all the samples arrayed in the slides for quantification. Values are expressed as means ± SEM. *p < 0.05, compared with normal brain tissues; ^#^*p* < 0.05, compared with grade I glioma tissues; one-way ANOVA with Tukey’s *post-hoc* test. Samples of normal brain tissue (*n* = 18), pilocytic astrocytoma (WHO grade I, *n* = 23), WHO grade I-II (*n* = 19), diffuse astrocytoma (WHO grade II, *n* = 85), anaplastic astrocytoma (WHO grade III, *n* = 27), and glioblastoma (WHO grade IV, *n* = 31). **e** Representative AEC-based AMOG immunostained images in normal human brain tissues and different grades of human glioma tissues. Photographs were taken with a digital microscope. Scale bars, upper panels 500 μm, middle panels 200 μm, and lower panels 20 μm

A tissue microarray was subjected to immunohistochemical staining of AMOG on normal brain tissue (both tumor-adjacent normal brain tissue and grey matter tissue from normal brain) and glioma tissues of different WHO grades. A light microscopic image of the AMOG-immunostained microarray was taken (Fig. 1a), and the black-and-white image of each tissue sample was generated by a gel imaging system and Fluochem software (Fig. 1b). Each tissue sample was rated and pooled according to the grade classification (Fig. 1c). As compared to the normal group, AMOG immunostaining intensities were considerably lower in the glioma tissue groups (*p* = 0.23, 0.019, < 0.000, < 0.000, and < 0.000 for grades I, I-II, II, III and IV, respectively). Intensities decreased from grade I to IV (*p* = 0.28, < 0.007 and < 0.004, respectively, versus grade I glioma; Fig. 1d). Representative immunohistochemical images of AMOG in normal human brain and different grades of human glioma tissues are shown at different magnifications (Fig. 1e). In summary, AMOG expression decreases with increasing tumor grade.

### AMOG siRNA increases L1 expression and reduces cell senescence and apoptosis in glioma cells

**Fig. 2:**
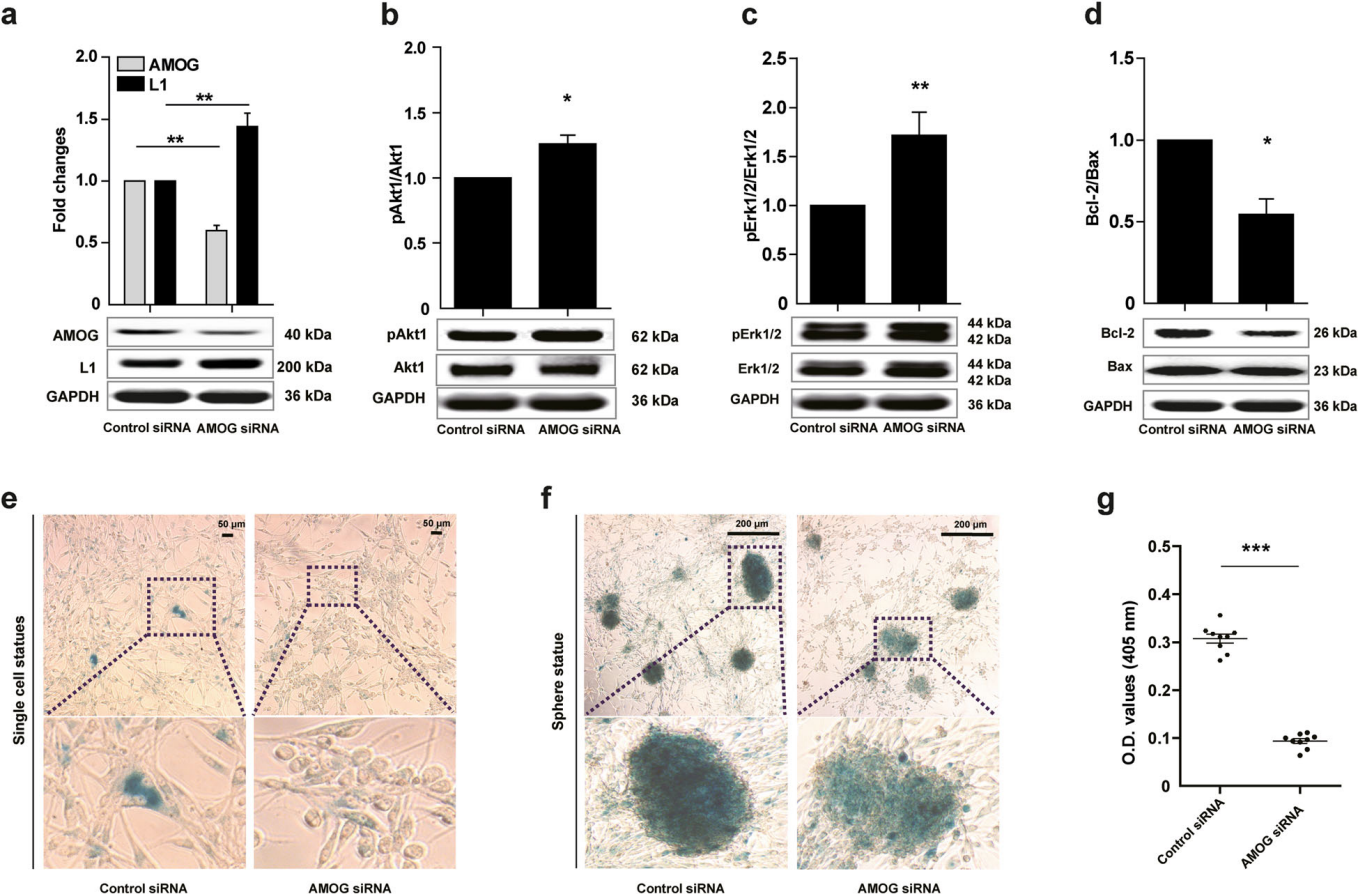
Reduction of AMOG expression affects L1-mediated apoptosis and senescence in correlation with signal transduction molecules in glioma U-87 MG cells. U-87 MG cells were transfected with either Control siRNA or AMOG siRNA. Western blot analysis for determining the levels of AMOG and L1, and L1 downstream signaling pathway proteins. Expression of AMOG and L1 (**a**), Akt1 and Erk1/2 phosphorylation levels (**b**, **c)**, as well as the ratio of Bcl-2/Bax (**d**) in U-87 MG cells in response to either Control siRNA or AMOG siRNA (10 nM). Values represent means ± SEM. (* *p* < 0.05 and ** *p* < 0.01 versus Control siRNA, two-tailed paired independent Student’s *t*-test, *n* = 4). **e-f** Since U-87 MG cells tend to form clusters before becoming confluent X-Gal staining in the single cell state and in clusters are shown after treatment with AMOG siRNA or Control siRNA. OD values show X-Gal staining. Representative photographs of single cells (**e**) and cell clusters (**f**) are shown for X-Gal staining. Scale bars = 50 and 200 μm, respectively. **g** Quantification of X-Gal staining of U-87 MG cells. OD values are expressed as means ± SEM. (*p < 0.05, **p < 0.01, and ***p < 0.001, versus Control siRNA, two-tailed paired independent Student’s *t*-test, triplicates of three independent experiments)

Erk and Akt signaling pathways contribute to cell migration, metastasis, senescence, and apoptosis in human glioma cells [18]. Bax and Bcl-2 are master regulators of apoptosis promotion and inhibition, respectively, and they are also regulated by the Erk and Akt. We then asked whether AMOG siRNA would affect L1 expression via signaling these molecules. AMOG siRNA reduced AMOG expression (*p* = 0.0041) and increased L1 expression (*p* = 0.0029) in comparison to the Control siRNA group in U-87 MG cells (Fig. 2a). Akt1 and Erk1/2 phosphorylation levels were increased in the AMOG siRNA group (*p* = 0.0154 for pAkt1/Akt1 levels, and *p* = 0.0002 for pErk1/2/Erk1/2 levels, versus Control siRNA; Fig. 2b, c). AMOG siRNA decreased the ratio of Bcl-2/Bax (*p* = 0.0173 versus Control siRNA, Fig. 2d). Taken together, reduction of AMOG expression increases L1 expression, in parallel with elevated levels of pErk and pAkt, and AMOG decreases the Bcl-2/Bax ratio in parallel with increased apoptosis.

To investigate whether reduction of AMOG expression leads to cell senescence, β-galactosidase activity was measured. After challenging with AMOG siRNA or Control siRNA for 48 h, U-87 MG cells were evaluated via the β-galactosidase X-Gal substrate. β-galactosidase activity was reduced in the group treated with AMOG siRNA as compared with the Control group as seen with single cells (Fig. 2e) and cell aggregates (Fig. 2f). Optical density values in the AMOG siRNA group were lower than in the Control siRNA group (*p* < 0.0001 versus Control siRNA, Fig. 2g), indicating that reduction of AMOG expression ameliorates glioma cell senescence in vitro.

### L1 siRNA increases AMOG expression in three glioma cell lines

**Fig. 3:**
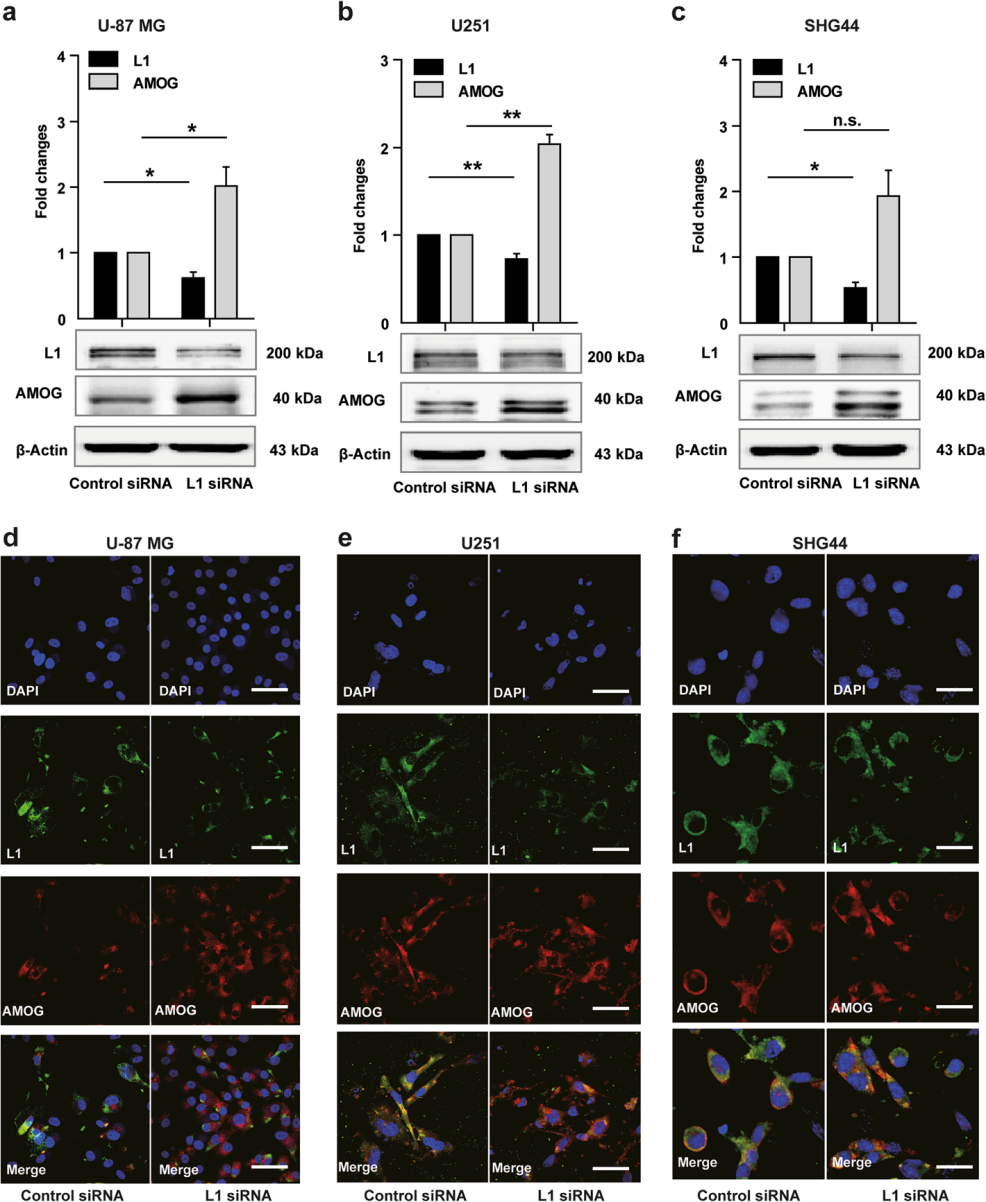
Reduced L1 expression leads to elevated AMOG expression. **a-c** Western blot analysis of L1 and AMOG expression in U-87 MG (**a**), U251 (**b**), and SHG44 (**c**) cells after treatment with L1 siRNA at 5 nM. Mean values ± SEM from 4 independent experiments are presented. (*p < 0.05, **p < 0.01, *** *p* < 0.001 vs. Control siRNA, independent Student’s *t*-test). **d-f** Representative images of double immunofluorescence staining of L1 and AMOG in response to L1 siRNA at 5 nM in U-87 MG (**d**), U251 (**e**) and SHG44 (**f**) cells. Scale bars = 50 μm

Since AMOG siRNA treatment increases L1 expression and reduces cell senescence, we asked whether L1 affects AMOG expression. As expected, L1 expression was reduced in U-87 MG, U251 and SHG44 cells treated with L1 siRNA (*p* = 0.023 in U-87 MG cells, *p* = 0.002 in U251 cells, and *p* = 0.043 in SHG44 cells, versus control siRNA; Fig. 3a, b, c). This treatment enhanced AMOG expression (*p* = 0.017 in U-87 MG cells, *p* = 0.01 in U251 cells, and *p* = 0.11 in SHG44 cells, versus control siRNA; Fig. 3a, b, c). Similar results for L1 and AMOG were obtained with 5, 10 and 20 nM L1 siRNA (Additional file 1: Figure S1a, b, c). In contrast to U-87 MG cells which shows one band for AMOG by Western blot analysis, two bands were detected in U251 and SHG44 cells. The bands between 45 kDa and 65 kDa represent the glycosylated and non-glycosylated forms of ATP1B2. A recent study [19] confirms our findings, showing two bands of AMOG between 35 and 70 kDa, representing both glycosylated and unglycosylated isoforms of AMOG in HEK293 cell.

In addition to Western blot analysis, double immunofluorescence staining of AMOG and L1 was performed in response to 5, 10 and 20 nM L1 siRNA application, which indicates that L1 siRNA increases AMOG expression (Fig. 3d, e, f, and Additional file 2: Figure S2a, b, c).

### L1 siRNA affects apoptosis-related signaling pathways in glioma cells

**Fig. 4:**
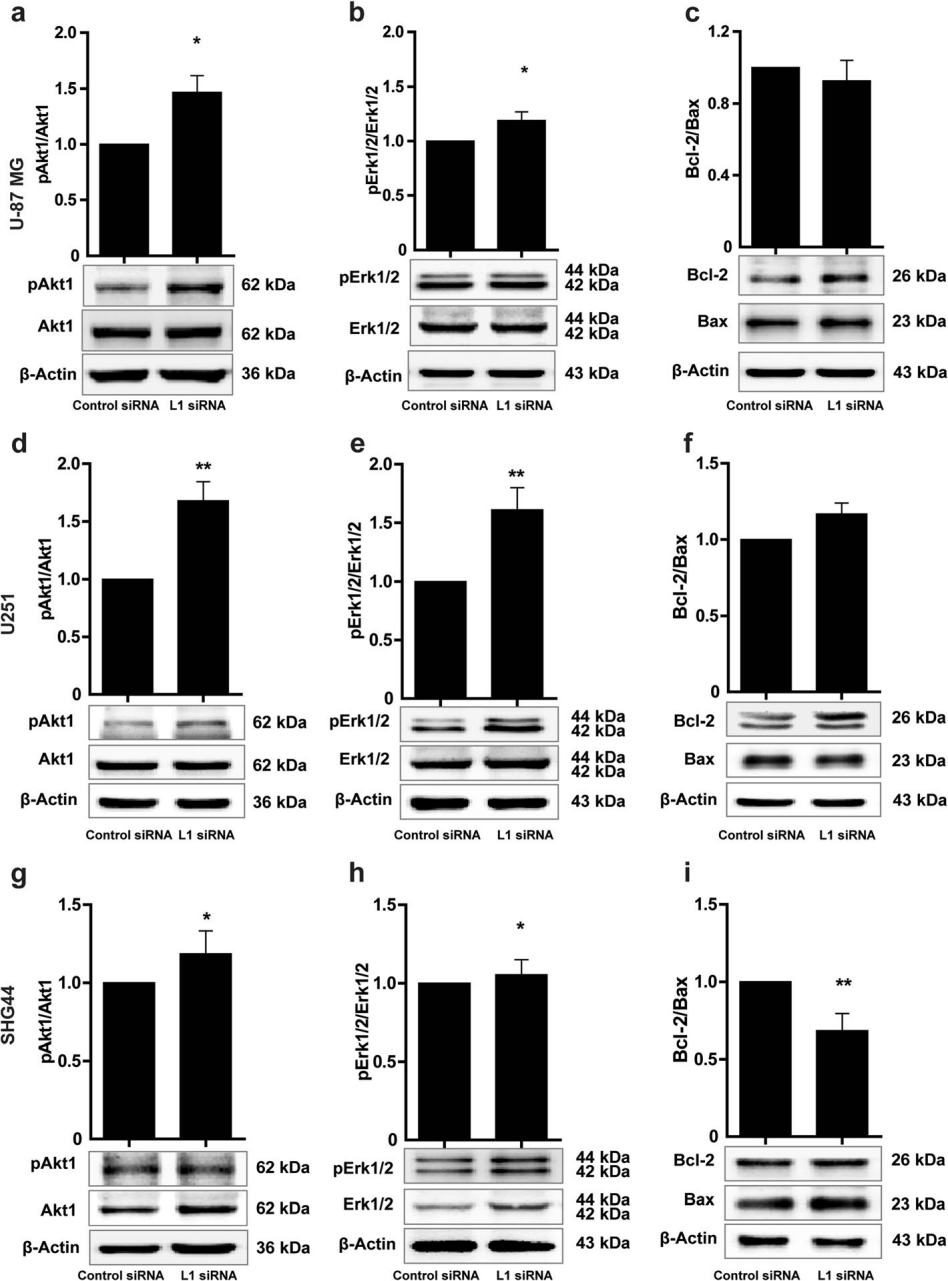
Influence of L1 siRNA on apoptosis-related signaling pathways in glioma cells. Western blot analysis of expression levels of phosphorylated Akt1 (**a, d, g**) and phosphorylated Erk1/2 (**b, e, h**), and ratio of Bcl-2/Bax (**c, f, i**) of U-87 MG, U251 and SHG44 cells after treatment with L1 siRNA. Mean values ± SEM are from 4 independent experiments; one-way ANOVA with Tukey’s *post-hoc* test. *p < 0.05 and **p < 0.001 versus Control siRNA group

Since signaling pathways are important features of a cell to influence cellular behavior, such as, for instance, migration, survival, senescence, and tumorigenesis, we tested paradigmatic such as pErk and pAkt, Bcl-2 and Bax. To this aim, L1 siRNA was applied to the glioma cells and Western blot analysis was performed to assay for these signal transducers. Compared to the Control siRNA group, L1 siRNA increased levels of pAkt1 and pErk1/2 (for U-87MG cells, *p* = 0.038 in Fig. 4a for pAkt1 levels, and *p* = 0.016; in Fig. 4b for pErk1/2 levels. For U251 cells, *p* = 0.003 in Fig. 4d for pAkt1 levels and *p* = 0.002 in Fig. 4e for pErk1/2 levels. For SHG44 cells, *p* = 0.045 in Fig. 4g for pAkt1 levels, and *p* = 0.039 in Fig. 4h for pErk1/2 levels; all *p* values refer to the Control siRNA). Interestingly, L1 siRNA did not affect the Bcl-2/Bax ratio (*p* = 0.886 versus Control siRNA for treatment of U-87 MG cells, Fig. 4c; *p* = 0.548 versus Control siRNA for treatment of U251 cells Fig. 4f). However, the Bcl-2/Bax ratio in SHG44 cells was considerably reduced by treatment with 5 nM L1 siRNA (*p* = 0.002 versus Control siRNA, Fig. 4i). To analyze whether these regulatory patterns on apoptosis**-**related signaling pathways would be different with higher concentrations of L1 siRNA, cells were treated with 10 and 20 nM of L1 siRNA for 48 h. Western blot analysis showed that suppressing L1 expression increased pAkt1 levels for in U-87 MG cells (*p* = 0.48 and *p* = 0.005 with 10 and 20 nM L1 siRNA, respectively, versus dose-matched Control siRNA, Additional file 3: Figure S3a). L1 siRNA at 10 and 20 nM for U251 and SHG44 cells reduced pAkt1 levels when compared to Control siRNA (*p* = 0.917 and *p* = 0.0487, respectively, in U251 cells; *p* = 0.832 and *p* = 0.002, respectively, in SHG44 cells; Additional file 3: Figure S3d, g). pErk1/2 levels showed a similar trend as seen for pAkt1 (Additional file 3: Figure S3b, e, h). The Bcl-2/Bax ratio was reduced in response to 20 nM of L1 siRNA (Additional file 3: Figure S3c, f, i). Thus, reduction of L1 expression reduces the Bcl-2/Bax ratio, which is characteristic of apoptosis. The combined results indicate that L1 inversely regulates AMOG expression and contributes to reduction of apoptosis mainly via Erk and Akt signaling.

### L1 reduces AMOG expression and suppresses apoptosis in glioma cells

**Fig. 5:**
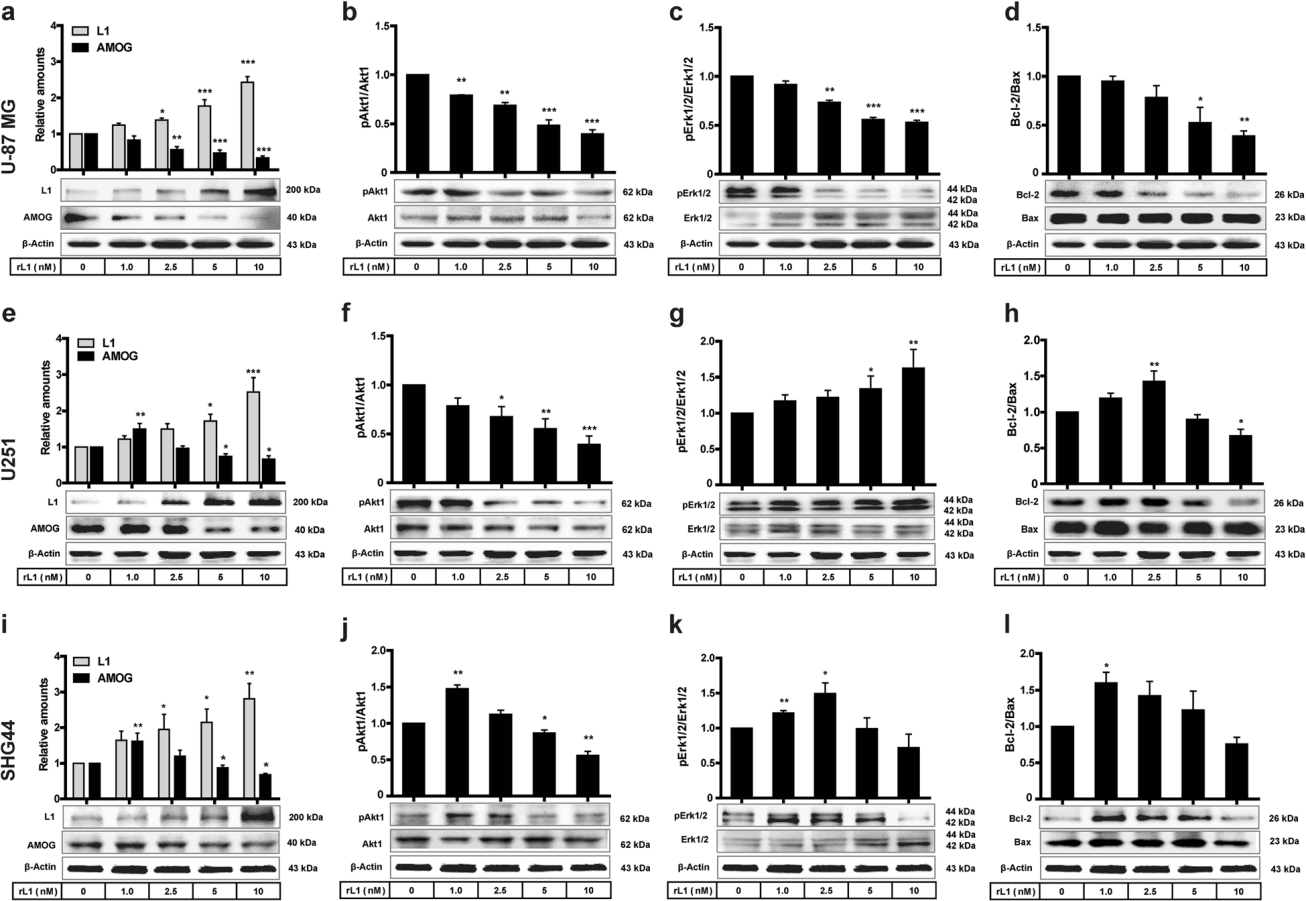
L1 reduces AMOG expression and suppresses apoptosis . Western blot analysis of L1 and AMOG (**a, e, i**), phosphorylation levels of Akt1 (**b, f, j**) and Erk1/2 (**c, g, k**), and ratio of Bcl-2/Bax (**d, h, l**) in U-87 MG, U251 and SHG44 cells after treatment with recombinant L1 (rL1) at concentrations between 0 to 10 nM. Mean values ± SEM are from 4 independent experiments; one-way ANOVA with Tukey’s *post-hoc* test. *p < 0.05, **p < 0.01, and ***p < 0.001 versus the Control siRNA group

Treatment of cells with recombinantly expressed extracellular domain of L1 (rL1) dose-dependently increased L1 levels in U251 and SHG44 cells (1, 2.5, 5 and 10 nM, *p* = 0.145, *p* = 0.026, *p* < 0.000, and *p* < 0.000 respectively, versus Control siRNA), and dose-dependently reduced AMOG levels in U-87 MG cells (*p* = 0.140, *p* = 0.001, *p* < 0.000, and *p* < 0.000 for 1, 2.5, 5 and 10 nM, respectively, versus Control siRNA, Fig. 5a). Similarly, L1 levels in U251 and SHG44 cells were also dose-dependently up-regulated in comparison with Control siRNA (for 1, 2.5, 5 and 10 nM, *p* = 0.467, *p* = 0.111, *p* = 0.024, *p* < 0.000, respectively, versus Control siRNA in U251 cells, Fig. 5e; for 1, 2.5, 5 and 10 nM, *p* = 0.189, *p* = 0.028, *p* = 0.024, *p* = 0.001 respectively, versus Control siRNA in SHG44 cells, Fig. 5i). Administration of 1.0 nM rL1 to U251 and SHG44 cells increased AMOG levels (*p* = 0.001 in U251 cells, Fig. 5e; *p* = 0.003 in SHG44 cells, Fig. 5i), whereas AMOG expression was decreased with increasing concentrations of rL1 (for 2.5, 5, and 10 nM*, p* = 0.779, *p* = 0.049 and *p* = 0.018, respectively, versus Control siRNA in U251 cells, Fig. 5e; *p* = 0.291, *p* = 0.049 and *p* = 0.019 for 2.5, 5, and 10 nM, respectively, versus Control siRNA in SHG44 cells, Fig. 5i). rL1 reduced pAkt1 levels in a dose-dependent manner in both U-87 MG and U251 cells (for 1, 2.5, 5 and 10 nM, *p* = 0.008, *p* = 0.001, *p* < 0.000, *p* < 0.000, respectively, versus Control siRNA, Fig. 5b; *p* = 0.102, *p* = 0.021, *p* = 0.004, *p* < 0.000, for 1, 2.5, 5 and 10 nM, respectively, versus Control siRNA, Fig. 5f). In contrast, pAkt1 levels in SHG44 cell were dose-dependently increased at 1 and 2.5 nM of rL1 (*p* = 0.001 and *p* = 0.094, respectively), and thus reduced compared to control (for 5 and 10 nM, *p* = 0.033 and *p* = 0.001, respectively, versus Control siRNA, Fig. 5j). Similar to pAkt1 levels, pErk1/2 levels were also reduced by rL1 in U-87 MG cells (for 1, 2.5, 5 and 10 nM, *p* = 0.059, *p* = 0.001, *p* < 0.000, *p* < 0.000, respectively, versus Control siRNA, Fig. 5c). In addition, pErk levels in U251 cell were dose-dependently increased by rL1 (for 1, 2.5, 5 and 10 nM, *p* = 0.455, *p* = 0.332, *p* = 0.0145, and *p* = 0.005, respectively, versus Control siRNA, Fig. 5g). A similar pattern as for pAkt1 expression was seen for pErk1/2 in SHG44 cells, which peaked at 2.5 nM of rL1 (for 1, 2.5, 5 and 10 nM, *p* = 0.003, *p* = 0.031, *p* = 0.959, and *p* = 0.221, respectively, versus Control siRNA, Fig. 5k). The Bcl2/Bax ratio was reduced in response to rL1 treatment in U-87 MG cells at 2.5, 5, and 10 nM (*p* = 0.733, *p* = 0.168, *p* = 0.016, *p* = 0.006, respectively, versus Control siRNA, Fig. 5d). However, in U251 cells the ratio Bcl2/Bax was increased at 1 and 2.5 nM, and decreased at 5 and 10 nM (for 1, 2.5, 5 and 10 nM, *p* = 0.146, *p* = 0.003, *p* = 0.437, and *p* = 0.023, respectively, versus Control siRNA, Fig. 5h). Similarly, the ratio of Bcl2/Bax was increased at 1 nM and decreased at 2.5, 5 and 10 nM (for 1, 2.5, 5 and 10 nM*, p* = 0.013, *p* = 0.099, *p* = 0.438, and *p* = 0.057, respectively, versus Control siRNA, Fig. 5i).

### Over-expression of AMOG reduces L1 expression and apoptosis-related signaling pathways

**Fig. 6:**
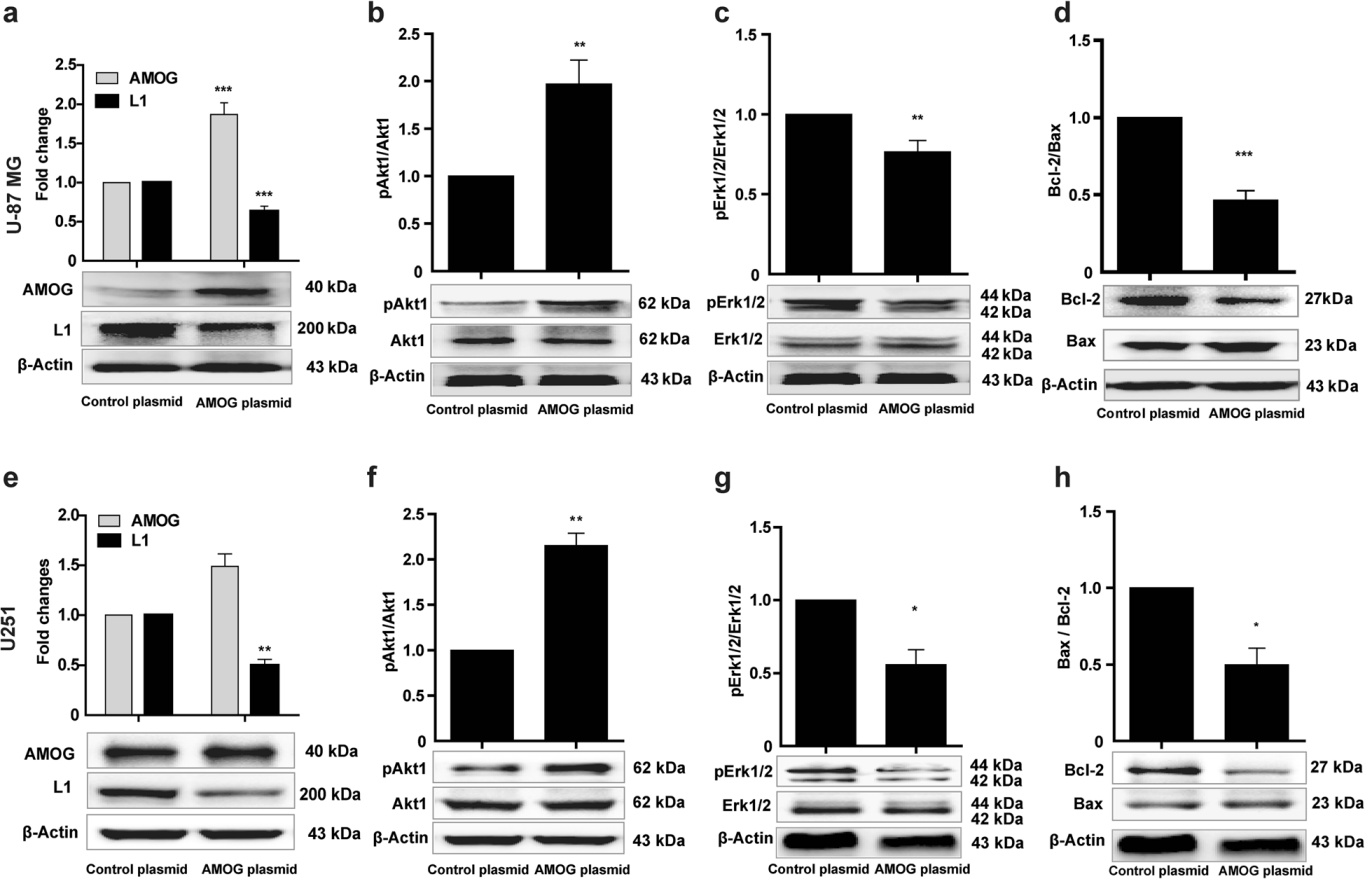
Over-expression of AMOG affects L1 expression and apoptosis-related signaling pathways. Western blot analysis of expression levels of L1 and AMOG (**a, e**), phosphorylated Akt1 (**b, f**) and Erk1/2 (**c** and **g**), and ratio of Bcl-2/Bax (**d, h**) in U-87 MG and U251 cells after transfection with an AMOG expression plasmid. Mean values ± SEM are from 3 independent experiments; independent Student’s *t*-test (*p < 0.05, **p < 0.01, and ***p < 0.001 versus the Control siRNA group)

To evaluate the role of AMOG on L1 expression and apoptosis, glioma cells were transfected with an AMOG encoding plasmid DNA. AMOG protein levels in U-87 MG and U251 cells were up-regulated (*p* < 0.000 in U-87 MG cells and *p* = 0.0588 in U251 cells, all *p* values refer to the Control plasmid DNA). In contrast, L1 levels were reduced when compared to the Control plasmid DNA group (*p* < 0.000 in U-87 MG cells, *p* = 0.0043 in U251 cells, Fig. 6a, e). Increased pAkt1 levels were seen in U-87 MG and U251 cells (*p* = 0.003 in U-87 MG cells and *p* = 0.0043 in U251 cells, versus Control plasmid, Fig. 6b, f). Yet, levels of pErk1/2 were reduced in both cell lines in comparison to the Control plasmid group (*p* = 0.009 in U-87 MG cells, *p* = 0.0435 in U251 cells, Fig. 6c, g). Bcl-2/Bax ratios were reduced in response to AMOG over-expression when compared to the Control plasmid group (*p* < 0.000 in U-87 MG cells and *p* = 0.0199 in U251 cells, Fig. 6d, h). Of note, over-expression of AMOG in SHG44 cells led to their failure to adhere to the cell culture dish, thereby evoking cell death. The combined results suggest that AMOG is involved in glioma cell apoptosis by reducing L1 expression. These features again point to differences in the functional features of tumor cells.

## Discussion

Gliomas have challenged researchers for decades with minimal progress towards an effective treatment due to the complexity of their cellular components, diffuse invasiveness and capacity to escape therapies [20–25]. Glial tumor cells express abnormal levels of cell adhesion molecules, highly correlating with the malignancy of these tumors [26–28]. Accordingly, cell surface and extracellular adhesion molecules have been studied for the management of invasive gliomas that mediate multiple cellular/molecular interactions and pathways [4, 29–33]. For instance, treatment with chimeric antigen receptors targeting L1 in 6 children with metastatic neuroblastoma via genetically engineered cytolytic T-lymphocytes allowed one patient to experience prolonged survival with a limited disease burden [34]. A recent study reported that L1 plays an essential role in initiating metastatic colonization in the spreading of disseminated cancer cells on capillaries, resulting in activation of Yes-associated protein (YAP) and myocardin-related transcription factor [35]. While L1-related in tumor malignancy has been well studied, relatively little is known about AMOG’s functions in tumors, yet present knowledge indicates that AMOG is associated with glioma cell invasiveness and migration [12, 13]. Thus, an understanding of the functional relationship between L1 and AMOG in glial tumors may offer new opportunities to develop specific and effective therapies.

In a previous investigation [36], using a microarray with the same catalogue, but different batch number, we had immunofluorescently demonstrated highest L1 levels in III and IV grade human glioma tissues and a positive correlation between the level of L1 expression and glioma grade: higher grade gliomas express less L1 than the lower grade gliomas. In the microarray used in the present study, AMOG expression was lowest in III and IV grade human glioma tissues and inversely correlated with the degree of tumor malignancy. Combining the previous with the present data suggests that AMOG inversely correlates with L1 expression. Thus, lower AMOG levels correlate with higher L1 levels in glioma cells, increasing with malignancy, and correlating with cell proliferation, survival, migration, invasion, and inhibition of apoptosis [4, 26, 30, 36]. Reduced expression of AMOG has been correlated with enhanced malignancy [36, 37], and raised the question about links of reciprocal influences in expression. We now show that reduction of AMOG expression in cultured glioblastoma cells leads to an increase in L1 expression and a decrease in apoptosis. Furthermore, both molecules are involved in regulating signal transduction mechanisms pertaining to apoptosis via Akt and Erk activation. Notably, AMOG subserves two functions: on the one hand, it is a recognition molecule and, on the other hand, it is part of the Na, K-ATPase and comprises the regulatory subunit of an important ion pump that depends on its function on this subunit [13, 37]. Further experiments are needed to allow interpretations of the influence of this pump activity on tumor progression and L1 expression.

Cell senescence is a key process involved in preventing tumor growth, and cancer cells have been considered to lose the capacity to be senescent [38, 39]. Emerging evidence has progressively contributed to our understanding of the central role of senescence as one of the processes inhibiting tumorigenesis [40]. In the present study, we found that reduction of AMOG expression delays cell senescence in vitro, and low AMOG expression correlates with reduced cell death. At first sight, this finding would be difficult to explain since one would assume that reduction of this functionally pivotal pump activity would increase cell death because of the resulting disturbed homeostatic balance. However, one could argue that an abnormal ionic balance may activate a cellular emergency program that could prevent cell death at least on a short-term basis, which was measured in the culture paradigms used in this study. Another explanation could be that low expression of AMOG leads to a higher expression of L1 and since L1 promotes survival of neurons in vitro and in vivo [41–43], it is plausible to assume that a higher L1 expression would promote cell survival. Given a possible functional outcome of opposite survival mechanisms, we suggest that the L1 driven promotion of cell survival would be more powerful in the determination of cell survival than ionic imbalance. The contribution of other adhesion molecules in tumorigenesis [44–47] could yield insights into the interplay between L1 and AMOG expression. Furthermore, activation of Akt and Erk signaling pathways has been found to be essential for glioma cell proliferation and invasion, and is correlated with tumor progression [48–52]. We noticed that L1 exerted differential effects on Akt and Erk activation in the three glioma lines. We had observed that U87-MG cells release large amounts of L1’s extracellular domain, which can trans-homophilically interact with full length L1 at the tumor cell surface to initiate a series of functional events [15]. The endogenous soluble L1 from the tumor cell in combination with exogenously added L1 may reduce the activation Akt and Erk actication in U87-MG cells. In contrast, the less malignant SHG44 cells may generate less L1, so that the effects of exogenous L1 on Akt and Erk can be detected.

It will be interesting in the context of the relationship between L1 and AMOG to identify the receptors for AMOG in relation to the cognate receptors for L1. We suggest that the findings of the present study contribute to ongoing attempts to develop a molecularly targeted therapy for glioma management.

## Conclusions

In conclusion, we show that AMOG expression is reduced in glioma cells with increasing malignancy, correlating inversely with L1 expression, which increases in expression with increasing malignancy. Down-regulation of AMOG expression induces cell senescence, which appears to be counteracted by increased L1 expression, which has been shown to enhance neuronal cell survival [53]. The mechanisms by which AMOG and L1 regulate their interactions remain unknown. It will thus be important to identify the receptors for AMOG, also in relation to the cognate receptors for L1. In addition, further investigations on interrelationships between cell adhesion molecules in tumorigenesis will be important to pursue.

## Additional files

Additional file 1: **Figure S1.** Reduction of L1 expression increases AMOG expression. Western blot analysis of L1 and AMOG expression in U-87 MG (**a**), U251 (**b**), and SHG44 (**c**) cells after treatment with L1 siRNA at 10 and 20 nM. Mean values ± SEM are from 4 independent experiments. (*p < 0.05, **p < 0.01, *** *p* < 0.001 vs. Control siRNA, independent Student’s *t*-test). (TIF 2142 kb)

Additional file 2: **Figure S2.** Expression of AMOG in relation to L1. Representative images of double immunofluorescence staining of L1 and AMOG after treatment with L1 siRNA at 10 and 20 nM in U-87 MG (**a**), U251 (**b**) and SHG44 (**c**) cells. Scale bars = 50 μm. (TIF 3736 kb)

Additional file 3: **Figure S3.** Apoptosis-related signaling pathways are affected by reduced L1 expression. Expression levels of phosphorylated Akt1 (**a**, **d**, **g**) and phosphorylated Erk1/2 (**b**, **e**, **h**), and ratio of Bcl-2/Bax (**c**, **f**, **i**) in U-87 MG, U251 and SHG44 cells after treatment with either 10 or 20 nM L1 siRNA. Mean values ± SEM are from 4 independent experiments. (One-way ANOVA with Tukey’s *post-hoc* test. *p < 0.05, **p < 0.01 and ***p < 0.001 versus Control siRNA group). (TIF 2889 kb)

## Data Availability

The authors confirm that all data and materials are kept at Shantou University Medical College and please contact the corresponding author for all data requests.

## Abbreviations

Akt: Protein Kinase B
AMOG: Adhesion molecule on glia
ECL: Enhanced chemiluminescence
Erk: Extracellular signal-regulated kinases
L1: Neural cell adhesion molecule L1 (L1CAM)
pAKT: phosphorylated Akt
pErk: phosphorylated Erk
PI3K: Phosphoinositide 3-kinase
rL1: recombinant human L1CAM extracellular domain
RNAi: RNA interference
WHO: World Health Organization
YAP: Yes-associated protein
