# Supplementary figures and images for: Glioma malignancy is linked to interdependent and inverse AMOG and L1 adhesion molecule expression
Source: BMC Cancer. 2019 Sep 12;19:911. doi: 10.1186/s12885-019-6091-5 (PMC6739972; doi:10.1186/s12885-019-6091-5)

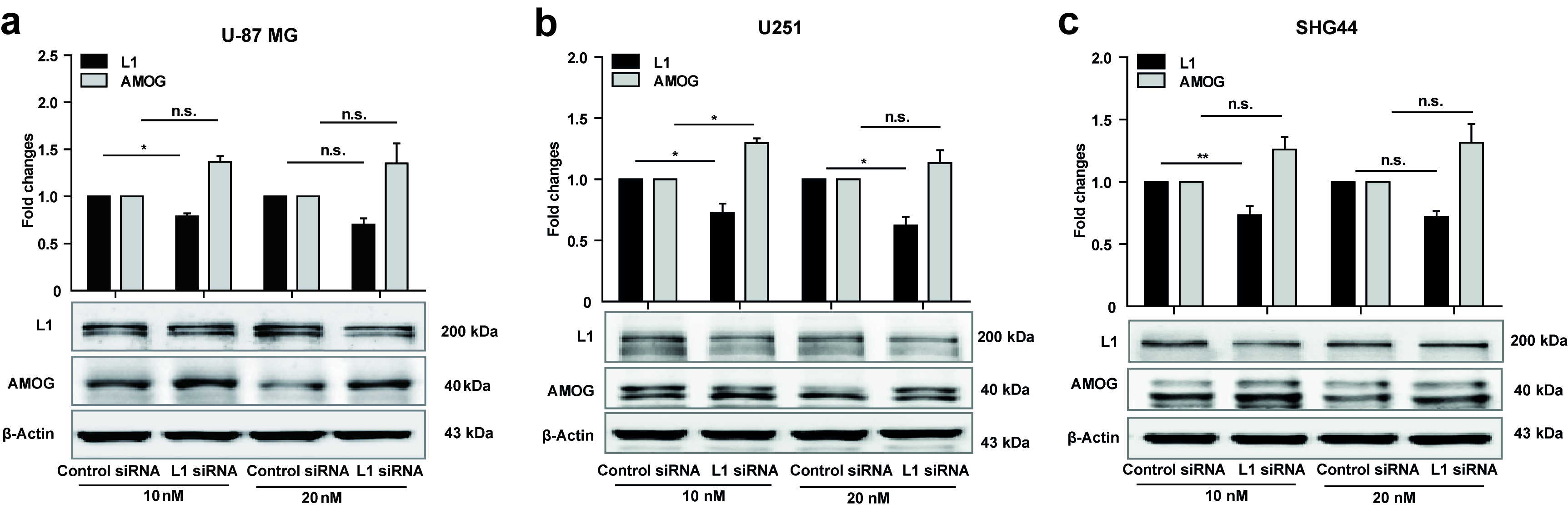

Supplement: Supplementary file 1 — Figure S1. Reduction of L1 expression increases AMOG expression. Western blot analysis of L1 and AMOG expression in U-87 MG (a), U251 (b), and SHG44 (c) cells after treatment with L1 siRNA at 10 and 20 nM. Mean values ± SEM are from 4 independent experiments. (*p < 0.05, **p < 0.01, *** p < 0.001 vs. Control siRNA, independent Student’s t-test). (TIF 2142 kb) [file 12885_2019_6091_MOESM1_ESM.tif]

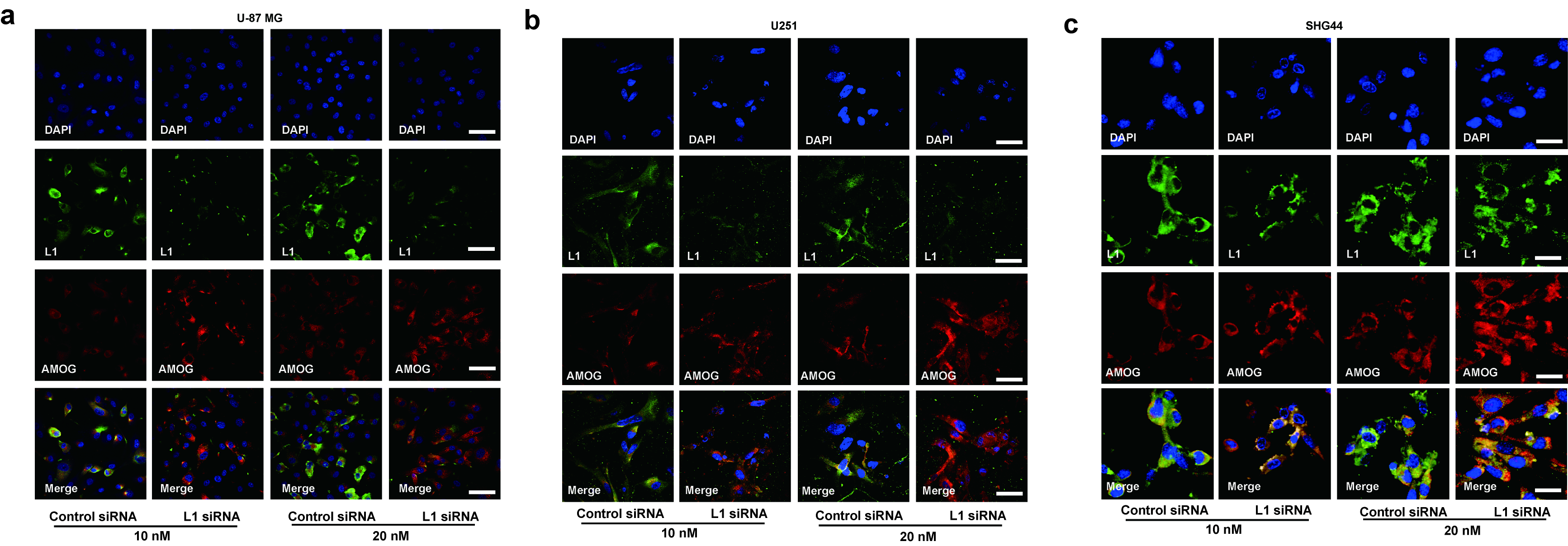

Supplement: Supplementary file 2 — Figure S2. Expression of AMOG in relation to L1. Representative images of double immunofluorescence staining of L1 and AMOG after treatment with L1 siRNA at 10 and 20 nM in U-87 MG (a), U251 (b) and SHG44 (c) cells. Scale bars = 50 μm. (TIF 3736 kb) [file 12885_2019_6091_MOESM2_ESM.tif]

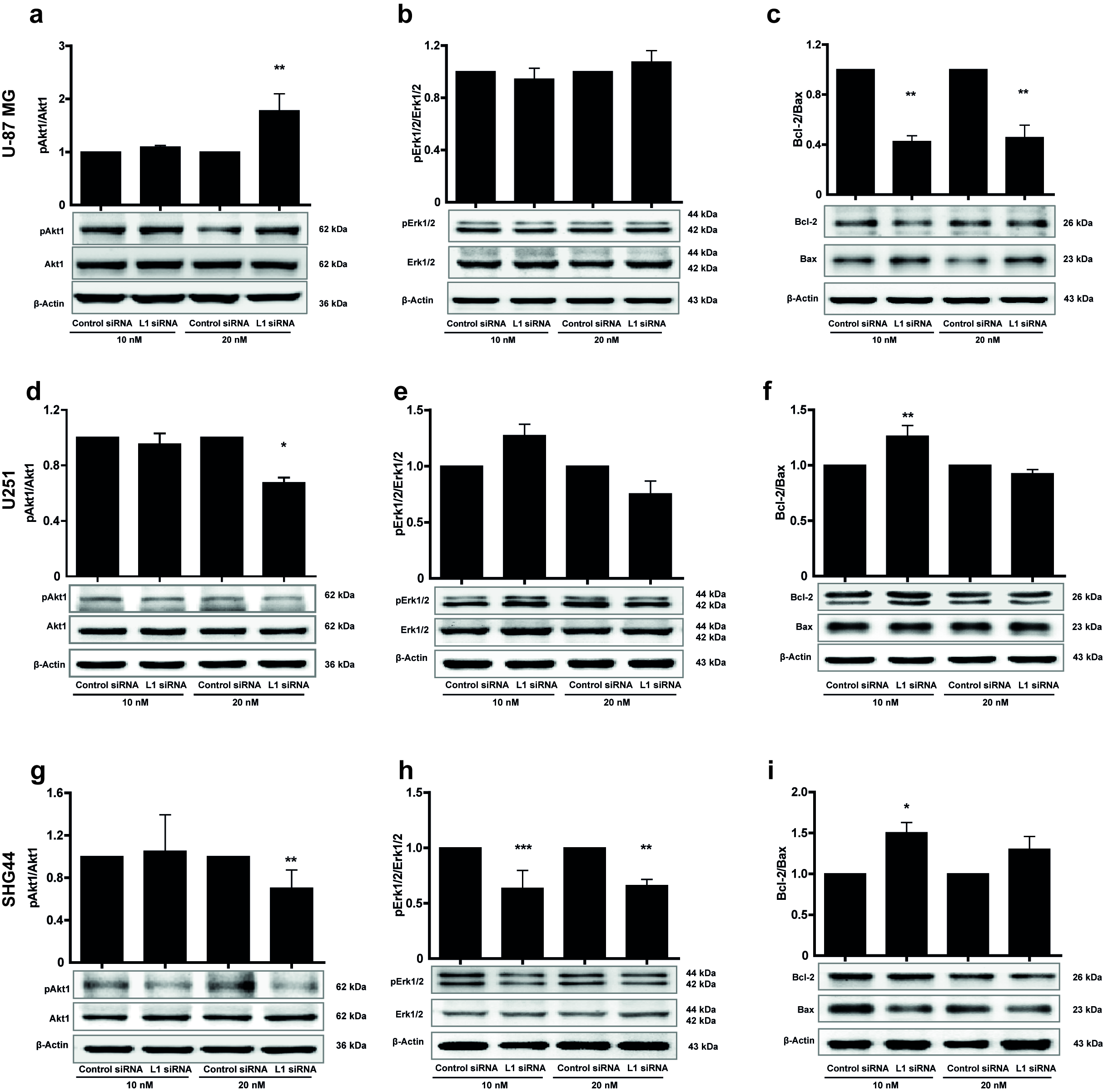

Supplement: Supplementary file 3 — Figure S3. Apoptosis-related signaling pathways are affected by reduced L1 expression. Expression levels of phosphorylated Akt1 (a, d, g) and phosphorylated Erk1/2 (b, e, h), and ratio of Bcl-2/Bax (c, f, i) in U-87 MG, U251 and SHG44 cells after treatment with either 10 or 20 nM L1 siRNA. Mean values ± SEM are from 4 independent experiments. (One-way ANOVA with Tukey’s post-hoc test. *p < 0.05, **p < 0.01 and ***p < 0.001 versus Control siRNA group). (TIF 2889 kb) [file 12885_2019_6091_MOESM3_ESM.tif]
